# Neutrophil Gelatinase-Associated Lipocalin in Synovial Fluid from Horses with and without Septic Arthritis

**DOI:** 10.3390/ani13010029

**Published:** 2022-12-21

**Authors:** Stine Jacobsen, Camilla Drejer Mortensen, Elisabeth Alkærsig Høj, Anne Mette Vinther, Lise Charlotte Berg, Ditte Marie Top Adler, Denis Verwilghen, Gaby van Galen

**Affiliations:** 1 Department of Veterinary Clinical Sciences, University of Copenhagen, DK-2630 Taastrup, Denmark; 2 BioPorto Diagnostics A/S, DK-3150 Hellerup, Denmark; 3 Sydney School of Veterinary Science, University of Sydney, Camperdown, Sydney, NSW 2050, Australia

**Keywords:** joint disease, inflammation, arthritis, synovitis, horse, equine, NGAL, LCN2, lipocalin-2, neutrophil gelatinase-associated lipocalin

## Abstract

**Simple Summary:**

Early detection of joint infection in horses is paramount, as continued infection may lead to degradation of articular structures and result in chronic lameness, sometimes of a severity that necessitates euthanasia for animal welfare reasons. Veterinarians use various diagnostic tests to establish the diagnosis, none of which have proven to ultimately be fully reliable. In humans, it has been shown that the biomarker neutrophil gelatinase-associate lipocalin (NGAL) has a very high diagnostic accuracy for joint infection. The purpose of this study was to evaluate the diagnostic potential of NGAL for the identification of joint infection in horses. NGAL was measured in 177 joint fluid samples from 152 horses suspected of joint infection. The results showed that NGAL was a very accurate diagnostic tool in horses and allowed the monitoring of the response to treatment. In the future, NGAL measurements may allow very early detection, and hence prompt instigation of therapy, of joint infection in horses—with presumed improvement of the prognosis of these patients.

**Abstract:**

Neutrophil gelatinase-associated lipocalin (NGAL) has been suggested to be a highly sensitive and specific marker of joint infection in humans. The aim of the study was to investigate NGAL concentrations in synovial fluid (SF) from horses with septic synovitis, horses without septic synovitis, and horses with uncertain status. NGAL was measured in 177 admission samples obtained from 152 horses. From a subset of horses (*n* = 35), additional samples obtained sequentially over the course of treatment were available. Concentrations of NGAL were significantly higher in septic synovitis (*n* = 47 samples) than in samples classified as non-septic (*n* = 103) or samples with uncertain status (*n* = 27), with median NGAL concentrations in the three groups being 1236, 16.8, and 266.4 µg/L, respectively. NGAL discriminated nearly perfectly between septic and non-septic (area under the receiver operating characteristic curve 0.98, 95% confidence interval 0.95–1.00). The optimal cut-off value for maximal sensitivity (87.2%) and specificity (75.0%) to discriminate septic samples from those with uncertain status was 444.6 µg/L, with an area under the receiver operating characteristic curve of 0.85 (95% confidence interval 0.74–0.93). Concentrations declined over time in horses undergoing treatment. NGAL is a novel biomarker that seems to have great potential for identifying septic synovitis and for monitoring the response to treatment of synovial infection in horses.

## 1. Introduction

Early recognition of septic synovitis in horses and prompt treatment are of the utmost importance, as delays in treatment may be life or career limiting [1,2,3]. Once established, septic synovitis may lead to systemic and local signs of inflammation such as fever, joint effusion and peri-capsular swelling, pain on palpation or manipulation, and heat in the tissues surrounding the synovial structure. In adult horses, septic synovitis results in lameness, while foals may be remarkably sound or show minimal lameness, even with advanced septic changes [4]. However, the diagnostic challenge consists of identifying synovial bacterial contamination or sepsis before an infection is fully established and clinical signs are apparent in order to best counteract the intrasynovial degenerative changes that result from the onsetting inflammation.

Relying on the bacterial culture of synovial fluid (SF) for diagnosis of septic synovitis is not ideal for two reasons; culturing takes a couple of days at least, and a significant percentage of false-negative results are to be expected—with growth rates ranging from 45 to 85% [5,6]. In a recent study, 48% of SF cultures from horses with confirmed septic synovitis were negative [7]. Most commonly, synovial sepsis is therefore diagnosed by the analysis of total and differential leukocyte counts, and total protein (TP) concentration in SF, where total leukocyte count (WBC) >30 × 10^9^/L, differential counts with >90% neutrophils, and TP concentration >30 g/L are highly suggestive of a septic process. Under certain circumstances these biomarkers may, however, yield inconclusive results. This may, for example, be in the early phases of joint contamination or septic synovitis of traumatic origin, in iatrogenic septic synovitis after intraarticular steroid injection, or in certain types (E and P, i.e., types involving bone) of septic synovitis in foals, where one or more of the three markers may remain below cut-off, thus obscuring interpretation of results [5,6,8]. Diagnostic and therapeutic procedures such as arthrocentesis, intraarticular injection of drugs, and arthroscopy have been shown to lead to increased leukocyte counts and TP concentration in SF—sometimes to levels encountered in septic synovitis [9,10,11,12]. Such treatment-induced increases in biomarker levels in SF may mask response to treatment and lead to the erroneous conclusion that infection is ongoing, which substantially hampers their usefulness in the monitoring of treatment efficacy.

Neutrophil gelatinase-associated lipocalin (NGAL), also known as lipocalin-2, human neutrophil lipocalin, 24p3, p25, migration-stimulating factor inhibitor, human neutrophil lipocalin, α1-microglobulin-related protein, siderocalin, or uterocalin, is a well-characterized protein which is released from neutrophil granulocytes, renal tubular cells, hepatocytes, adipocytes, and other cell types, as well as in tissues exposed to microorganisms [13]. NGAL is involved in many physiological and pathological processes, e.g., apoptosis, transport of fatty acids, metabolic homeostasis, and modulation of inflammation [13]. In infectious diseases, NGAL exerts bacteriostatic effects through the facilitation of intracellular iron sequestration in the host [14]. Bacteria require iron for their growth and respond to low iron levels by secretion of siderophores, which are small, high-affinity iron-scavenging compounds that transport iron into the microbe. NGAL possesses a ligand binding cavity that interact with siderophores. Through this binding, iron-laden siderophores are transported into the host cells, thereby limiting iron availability [15,16].

In humans, it has been known for 30+ years that serum concentrations of NGAL increase in inflammatory and infectious diseases. This was shown in infants with infections such as sepsis, osteomyelitis or deep soft tissue infections [17], children with bacterial and viral infections [18], and adults with airway infections [19]. More recently, NGAL has been described in inflammatory conditions in horses. Increased blood levels of NGAL were demonstrated in horses with naturally occurring and experimental inflammation [20,21,22], and a recent study reported increased concentrations of NGAL in SF after experimental induction of joint inflammation (by intraarticular injection of lipopolysaccharide [LPS] or local analgesics) and in a small (n = 10) group of horses with naturally occurring septic synovitis [20]. Concentrations of NGAL in SF increased 343-fold in response to intraarticular injection of LPS and were approximately 350 times higher in horses with septic synovitis than in SF from healthy horses [20], suggesting an excellent diagnostic potential. This has been corroborated in four fairly recent studies investigating NGAL in SF from people with periprosthetic joint infection (PJI) after hip or knee replacement surgery [23,24,25,26], where SF NGAL concentrations were substantially higher in patients with PJI than in healthy controls and patients with aseptic implant failure. In all studies, SF NGAL had excellent discriminatory capacity with high sensitivity and specificity.

To obtain more robust data on the diagnostic accuracy of NGAL in equine SF, the aim of this study was to measure NGAL in SF in horses with suspected septic synovitis.

## 2. Materials and Methods

Synovial fluid samples were available from 152 horses. Horses had 1–4 synovial structures sampled (1 = 130 horses, 2 = 20 horses, 3 = 1 horse, and 4 = 1 horse), yielding 177 admission samples. Samples were available from a biobank and had been collected routinely at admission as part of the diagnostic work-up of patients admitted to the Large Animal Teaching Hospital, University of Copenhagen between the 1st of January 2016 and the 31st of December 2018. Reasons for obtaining the samples were the presence of a wound over a synovial structure (with possible synovial penetration), suspicion of septic arthritis of hematogenous, iatrogenic or idiopatic origin, and suspicion of a spread of infection from the peri-synovial infectious process. Synoviocentesis was performed with 19- to 21-gauge needles after aseptic preparation of the injection site and SF immediately transferred to tubes containing ethylenediaminetetraacetic acid (BD Vacutainer; Becton Dickinson A/S, Albertslund, Denmark). Samples were stored at 5 °C until processing. TP content was determined using refractometry. A WBC count was obtained within 1–60 h by manual counting in a hemocytometer, and differential counts were performed in Hemacolor-stained cytospin preparations by counting 200 cells. The remainder of the sample was centrifuged at 1200 g for 10 min and stored at −20 °C until NGAL analysis, which took place in March 2019.

Based on the attending clinician’s decision, SF samples were submitted for bacteriology from 17 horses. Samples were placed in blood culture bottles (BD Bactec; Becton Dickinson A/S, Albertslund, Denmark) and treated according to the manufacturer’s recommendations. This entails 24 h of pre-culturing at 37 °C before shipping to a commercial laboratory, where aerobic and anaerobic culture as well as antimicrobial sensitivity testing were performed.

From a subset of the included horses (*n* = 35), sequential SF samples were available. These samples were obtained between 1 and 34 days of admission as part of the hospital routine to monitor response to therapy. Horses had 1–5 follow-up samples obtained.

Based on the results from the clinical and diagnostic workup on the day of admission, horses were divided into three groups: (1) septic synovitis, defined as a WBC count >30 × 10^9^/L leukocytes and one (or both) of the following: TP >30 g/L and neutrophil granulocyte percentage (neutrophil%) >90. Horses were also defined as septic if there was a positive bacteriology result, (2) non-septic, defined as WBC <5 × 10^9^/L leukocytes, and (3) uncertain, defined as WBC >5 and <30 × 10^9^/L leukocytes.

Concentrations of NGAL were assessed using a commercially available enzyme-linked immunosorbent assay (ELISA) based on porcine antibodies (Horse NGAL ELISA No. 49; BioPorto Diagnostics A/S, Hellerup, Denmark). The ELISA has been validated and found reliable for equine use by our research group [27]. The ELISA was performed according to the manufacturer’s instructions. Absorbance was read at 450 nm (reference, 620) in an ELISA reader (Multiskan EX; ThermoFisher Scientific, Hvidovre, Denmark) with Ascent Software version 2.6 to EMS Reader MF (Thermo Labsystems, Philadelphia, PA, USA). By using a four-parameter logistic regression according to the manufacturer’s instructions, absorbances were converted to concentrations (pg/mL) in MyAssays (www.myassays.com accessed 18 December 2022). Concentrations were subsequently converted to μg/L.

Descriptive statistics were used for data presentation. Normality was assessed using the Kolmogorov–Smirnov test, which showed that none of the variables (WBC, neutrophil%, TP, NGAL concentration) had normal distribution; group comparisons were thus made using the non-parametric Kruskal–Wallis test and the post-hoc Dunn’s multiple comparisons test. The correlation between variables was assessed with linear regression. Receiver operating characteristic (ROC) curves were generated to determine the diagnostic value of NGAL to identify septic synovitis. The area under the curve (AUC) and 95% confidence intervals (CIs) were calculated. The optimal cutoff for NGAL as a diagnostic tool for septic synovitis was determined using the Youden index. Statistical analyses were performed with GraphPad Prism software (Version 9.4.1. (681); GraphPad Software Inc., San Diego, CA, USA) and MedCalc Statistical Software (Version 20.114; MedCalc Software Ltd., Ostend, Belgium).

## 3. Results

### 3.1. Horses

The age of horses ranged from 1 day to 22 years. For three horses, age was not recorded in the file. There were 23 (15.1%) foals (less than 6 months old). Horses were of 25 different breeds (for four horses, breed was not recorded in the file). The predominant breeds were warmbloods (*n* = 58, 38.2%), Icelandic horses (*n* = 23, 15.1%), and thoroughbreds (*n* = 16, 10.5%). There were 73 mares (48.0%), 53 geldings (34.9%), 23 stallions (15.1%), and three horses of unknown sex (1.9%).

Reasons for performing synoviocentesis were wound in proximity of synovial structure (*n* = 102), suspicion of hematogenous (*n* = 21), iatrogenic (*n* = 11) or idiopathic septic synovitis (*n* = 11), and suspicion of spread from the peri-articular infection to the nearby synovial structure (*n* = 7). There were 44 (28.9%) horses eventually diagnosed with sepsis in one or more synovial structures, while 81 (53.3%) horses were non-septic and 27 (17.8%) had uncertain status.

Depending on the final diagnosis; horses either received no medical treatment; were treated with NSAIDs alone; or were treated with a combination of NSAIDs, antimicrobials, and/or surgery (joint lavage and debridement, debridement of osteomyelitic focus, wound management).

### 3.2. Samples and Synovial Structures

Samples originated from 19 different synovial structures, where the majority were joints (*n* = 144, 81.4%) and the remainder were tendon sheaths (*n* = 28, 15.8%) and bursae (*n* = 5, 2.8%) (Table 1). There were 47 (26.6%) samples classified as septic, 103 (58.2%) as non-septic, and 27 (15.3%) as uncertain (Table 1). Bacteria were cultured in 10/17 (58.8%) samples, yielding one or more species identified in each sample: *Streptococcus* spp., Pantoea agglomerans, *Staphylococus* spp., *Actinobacillus* spp., Klebsiella (not further characterized), and *Acinetobacter* spp.

In horses with septic synovitis, WBC, neutrophil%, and TP in SF were median (range) 84.0 × 10^9^/L (30.2–180.6), 90.5% (56.0–100.0), and 45 g/L (14–86), respectively. In horses in the non-septic group WBC, neutrophil%, and TP in SF were median (range) 0.4 × 10^9^/L (0.0–4.9), 34.5% (1.5–87.0), and 14 g/L (0–70), respectively, and in horses with uncertain classification WBC, neutrophil%, and TP in SF were median (range) 10.0 × 10^9^/L (5.3–21.7), 77.0% (34.0–87.5), and 28 g/L (8–80), respectively.

### 3.3. NGAL Concentrations

**Figure 1:**
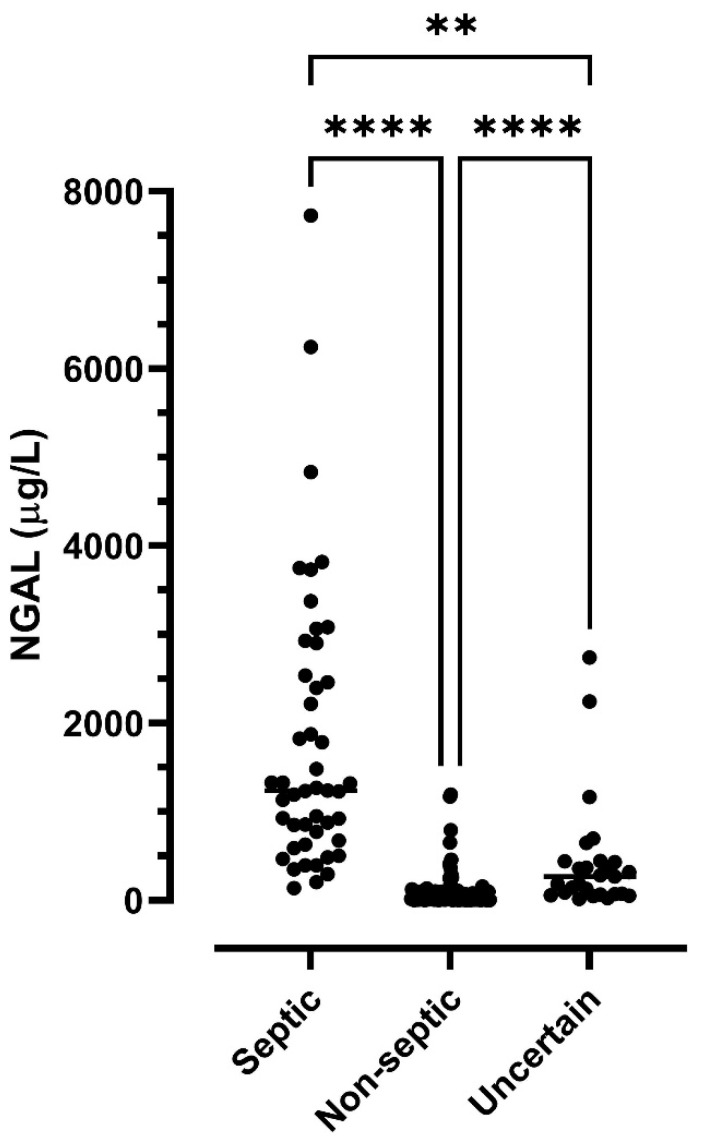
Concentrations of neutrophil gelatinase-associated lipocalin (NGAL) in synovial fluid samples from 152 horses suspected of septic synovitis. Samples were classified as septic (*n* = 47), non-septic (*n* = 103), or uncertain (*n* = 27) based on total leukocyte count, differential leukocyte counts, total protein concentration, and the results of bacteriology. Asterisks show significant differences between groups, ** *p* < 0.01; **** *p* < 0.0001.

Median concentrations of NGAL in SF in the septic, non-septic, and uncertain group were 1236, 16.8, and 266.4 µg/L, respectively; these concentrations were significantly different (overall *p* < 0.0001) with all group medians being significantly different pairwise (Figure 1).

**Figure 2:**
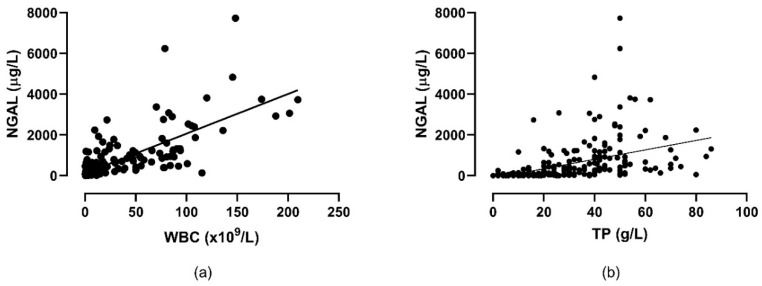
Correlation between concentrations of neutrophil gelatinase-associated lipocalin (NGAL) and (**a**) total leukocyte count (WBC) and (**b**) total protein (TP) in synovial fluid samples from 152 horses with and without septic synovitis.

Synovial fluid NGAL concentration correlated significantly with SF WBC (*p* < 0.0001. R^2^ = 0.57) and SF TP (*p* < 0.0001, R^2^ = 0.19) (Figure 2).

**Figure 3:**
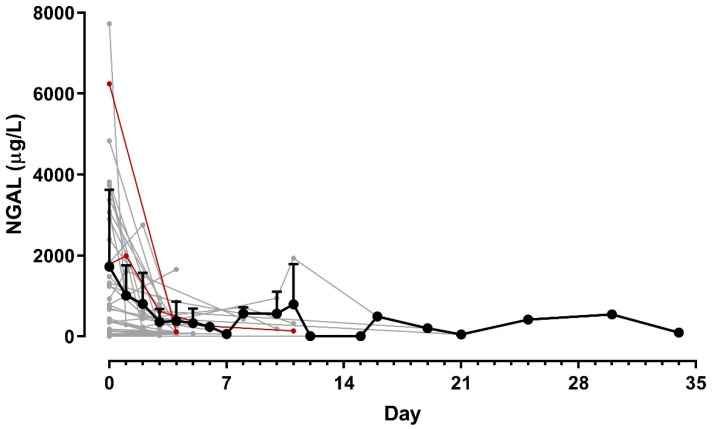
Concentrations of neutrophil gelatinase-associated lipocalin (NGAL) in synovial fluid obtained sequentially over the course of treatment of 35 horses suspected of septic synovitis. Samples were obtained at the attending clinician’s discretion, so with varying intervals, 1–34 days after initiation of treatment. Thin lines represent individual horses (grey = survivors [n = 33]; red = euthanized horses [*n* = 2]), the bold black line represents average of all horses, error bars are standard deviations.

In horses with sequentially obtained samples, SF NGAL concentrations declined in response to treatment (Figure 3). All but two of the 35 horses with sequential samples survived to discharge.

**Figure 4:**
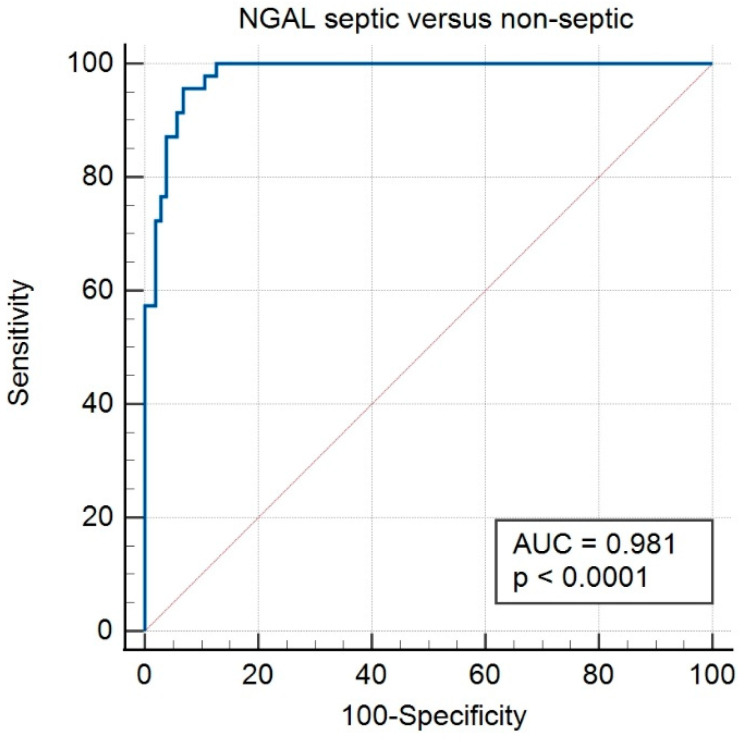
Receiver operating characteristic curve for diagnostic ability (differentiation of septic and non-septic) of neutrophil gelatinase-associated lipocalin (NGAL) in synovial fluid from horses. AUC = area under the curve.

To analyze the ability of NGAL to discriminate septic from non-septic, but also septic from uncertain and uncertain from non-septic, ROC curves were plotted (Figure 4, Table 2). The best threshold (cut-off) for distinguishing septic from non-septic was 293.6 µg/L, and the best threshold for distinguishing septic from uncertain was 444.6 µg/L (Table 2). Diagnostic strength (AUC) was very good to excellent (Table 2).

Using the septic versus non-septic cut-off of 293.6 µg/L would result in 12/27 (44.4%) samples in the uncertain group being classified as septic.

## 4. Discussion

NGAL demonstrated desirable diagnostic ability, with significantly higher concentrations in SF from horses with septic synovitis than non-septic and uncertain cases, and with excellent ability to discriminate septic from non-septic cases. Median concentration of NGAL in septic SF samples (1236 µg/L) corresponded roughly to those reported in SF from humans with PJI (1536.5–2007 µg/L) [23,26].

In horses, two studies have assessed NGAL in inflammation. These showed that serum NGAL concentrations were elevated in horses with abdominal disease characterized by inflammation (duodenitis-proximal jejunitis, colitis/typhlocolitis, and/or peritonitis) compared to horses with simple intestinal or strangulating obstructions [21] and also in horses with experimental and naturally occurring joint inflammation compared to healthy controls [20]. These studies also showed that NGAL is not only present in the systemic circulation, but that it can also be measured in peritoneal fluid [21] and SF [20]. Assessing NGAL locally in the inflamed joint was suggested to be more sensitive than serum measurements, and with its fast rise-and-fall pattern (concentrations in SF peaked 8 h after joint inflammation had been elicited by intraarticular injection of LPS), it was suggested that this analyte might be particularly well suited for the early diagnosis and monitoring of joint inflammation [20].

Concentrations of NGAL in SF were able to discriminate septic from non-septic with high accuracy (sensitivity, specificity, and AUC under the ROC curve of 95.7%, 93.2%, and 0.98, respectively). For differentiating septic joints from joints with uncertain status, specificity was substantially lower (75.0%), but sensitivity still acceptable (87.2%), which suggests that SF NGAL may be most suited for ruling out disease. The sensitivity and specificity of NGAL compares favorably with the diagnostic accuracy of other SF biomarkers. Recently, neutrophile granular enzymes lysozyme and myeloperoxidase were shown to have 80–100% sensitivity and specificity for differentiating SF with septic inflammation from aseptic inflammation or healthy SF [28]. The acute phase protein serum amyloid A was assessed in a study involving 62 horses presented with injured synovial structures and used to differentiate septic from non-septic SF; results showed that serum amyloid A had excellent sensitivity (0.93) and good specificity (0.77) [29].

The desirable characteristics of SF NGAL measurements determined on our horses are corroborated by five studies in humans, where NGAL was quantified in SF [23,24,25,26] or serum [30] in patients suspected of PJI after total knee or hip arthroplasty. All studies showed higher NGAL concentrations in patients with PJI than in patients without PJI. Two studies investigated the ability to differentiate aseptic failure or loosening of the implants from PJI, which is a major clinical challenge in arthroplasty surgery, and showed that SF [23] and serum [30] NGAL were able to differentiate these two conditions with good sensitivity (81.6–86.3%) and specificity (77.2–85.0%), and excellent AUC under the ROC curve (0.92 in both studies). The optimal cut-off between the two conditions was 152 µg/L in SF [23] and 105.1 µg/L in serum [30], which is slightly lower than the cut-off values determined in our horses (293.6 and 444.6 µg/L for differentiating septic from non-septic and septic from uncertain, respectively). Another two studies, which investigated differentiation between PJI and non-PJI, found the optimal SF NGAL cut-off concentrations to be 735.5 [26] and 762.8 µg/L [25]. Differences between species, assays, and differences in the pathogenesis of disease (for obvious reasons our data did not contain PJI cases) may potentially explain these variations in cut-off values. We have previously shown that NGAL concentrations in SF greatly exceed those in serum [20], and the body fluid analyzed will thus also affect cut-off values.

It is important to note that neither the present study, nor the previous studies on detection of PJI in humans were conducted according to the STARD criteria accepted for conducting and publishing studies on diagnostic accuracy (https://www.equator-network.org/reporting-guidelines/stard/, accessed 18 December 2022). Deviations from these criteria may affect estimated indices of diagnostic accuracy. For our study, samples were obtained from our biobank, and they may therefore not exactly represent the real diagnostic circumstances of clinical practice. Including a group of samples with uncertain sepsis status does, however, reflect the complexity encountered under clinical circumstances and gives a more comprehensive impression of diagnostic accuracy. Moreover, sensitivity and specificity are not affected by disease prevalence, and considering the uniformly positive results from our and all the other available studies on diagnostic accuracy of SF NGAL for identification of septic synovitis [23,24,25,26,30], it seems likely that the true accuracy is good to excellent. NGAL may thus be a valuable adjunct to current diagnostics.

It is important to remember that conditions other than septic synovitis may cause NGAL concentrations to increase in the systemic circulation. The protein originates from two major sources: neutrophils, where it is stored as a preformed molecule in the granules and may be released during inflammation, and epithelial cells (e.g., in the kidney, gastro-intestinal tract, uterus, liver, spleen, and various cancer cells), where de novo synthesis can be elicited in response to a variety of stimuli [13,31]. Systemic inflammation of any etiology, including aseptic inflammation, will cause increased NGAL concentrations in the systemic circulation, as demonstrated in horses after castration [22] or experimental IV LPS injection [22]. Acute kidney injury also results in an increase in NGAL in urine and serum/plasma [22,27,32,33], and endometritis and pregnancy has been shown to be accompanied by increased expression of NGAL in equine endometrial biopsies [34,35,36,37,38]. Assessing NGAL in the compartment of specific interest, e.g., SF for joint disease, peritoneal fluid for abdominal disease, and urine for renal disease, is likely to increase diagnostic specificity. Aseptic joint inflammation will give rise to increased SF NGAL concentrations, as shown in horses after intraarticular injection of local analgesics [20] and in humans with osteoarthritis, rheumatoid arthritis, ankylosing spondylitis, spondyloarthropathy, and psoriatic arthritis, but levels are substantially lower than in septic joints, which may help differentiate the two conditions [25,39,40].

A main limitation of the study and a limitation for the current clinical use of NGAL is the lack of clinically relevant assay formats for the detection of equine NGAL. ELISAs are mainly useful for the bulk analysis of samples in research, not for on-demand, single-sample, routine detection, and this limits the clinical use of this analyte at this point.

## 5. Conclusions

The diagnostic accuracy of SF NGAL was good to excellent in a hospital cohort of horses suspected of septic synovitis. Concentrations of NGAL declined in response to treatment, suggesting that NGAL may also be used to monitor the response to treatment of synovial infection. Taken together, these results suggest that NGAL could be a valuable adjunct to the current modalities available for diagnosing synovial sepsis. As ELISAs are mainly useful for measuring samples in bulk in research projects, the development of an assay that can be used on-demand and that has a short turnaround time is the prerequisite for future clinical use of NGAL.

## Figures and Tables

**Table 1:** Synovial samples (*n* = 177) obtained at admission from 152 horses suspected of septic synovitis. Samples were classified as septic, non-septic, or uncertain based on total leukocyte count, differential leukocyte counts, total protein concentration, and the results of bacteriology.

Synovial Structure	Number and Percentage of Samples	Classification
Tarsometatarsal joint	1 (0.6%)	Septic = 0
		Non-septic = 1
		Uncertain = 0
Carpal and tarsal sheaths	2 (1.1%)	Septic = 0
		Non-septic = 2
		Uncertain = 0
Extensor tendon sheaths (common digital extensor, extensor carpi radialis)	2 (1.1%)	Septic = 1
		Non-septic = 1
		Uncertain = 0
Scapulohumeral joint	2 (1.1%)	Septic = 1
		Non-septic = 1
		Uncertain = 0
Navicular bursa	2 (1.1%)	Septic = 0
		Non-septic = 2
		Uncertain = 0
Calcaneal bursa	3 (1.7%)	Septic = 2
		Non-septic = 1
		Uncertain = 0
Cubital joint	5 (6.5%)	Septic = 3
		Non-septic = 1
		Uncertain = 1
Lateral/medial femorotibial joints	7 (2.0%)	Septic = 1
		Non-septic = 5
		Uncertain = 1
Femoropatellar joint	10 (5.6%)	Septic = 3
		Non-septic = 5
		Uncertain = 2
Distal interphalangeal joint	16 (9.0%)	Septic = 0
		Non-septic = 12
		Uncertain = 4
Digital flexor tendon sheath	24 (13.6%)	Septic = 7
		Non-septic = 15
		Uncertain = 2
Carpal joints (radiocarpal and middle carpal)	27 (15.3%)	Septic = 5
		Non-septic = 19
		Uncertain = 3
Metacarpo- and metatarsophalangeal joints	31 (17.5%)	Septic = 15
		Non-septic = 10
		Uncertain = 6
Tarsocrural joint	45 (25.4%)	Septic = 9
		Non-septic = 28
		Uncertain = 8
Total	177 (100%)	Septic = 47
		Non-septic = 103
		Uncertain = 27

**Table 2:** Discriminatory ability of neutrophil gelatinase-associated lipocalin in synovial fluid for identification of three groups of horses: septic synovitis (*n* = 47 samples), non-septic (*n* = 103 samples), and uncertain status (*n* = 27 samples).

ROC Curve	Sensitivity (%)	Specificity (%)	Area under the Curve (95% Confidence Interval)	Cutoff Value (µg/L)	*p*-Value
Septic versus non-septic	95.7	93.2	0.981 (0.945–0.996)	293.6	<0.0001
Septic versus uncertain	87.2	75.0	0.849 (0.740–0.925)	444.6	<0.0001
Uncertain versus non-septic	88.9	69.9	0.853 (0.781–0.909)	52.8	<0.0001

## Data Availability

The data presented in this study are available on request from the corresponding author.
